# Autonomic and psychophysiological effects of a 13-week mindfulness-based intervention in university students

**DOI:** 10.3389/fpsyg.2026.1704398

**Published:** 2026-02-04

**Authors:** Juan Camilo Benítez-Agudelo, Eduardo Navarro-Jiménez, Vicente Javier Clemente-Suárez

**Affiliations:** 1 Departamento de Ciencias Sociales, Corporacion Universitaria de la Costa, Barranquilla, Colombia; 2 Faculty of Medicine, Health and Sports, Universidad Europea de Madrid, Villaviciosa de Odón, Madrid, Spain; 3 Universidad Simón Bolívar, Facultad de Ciencias de la Salud, Centro de Investigaciones en Ciencias de la Vida, Barranquilla, Colombia

**Keywords:** academic performance, heart rate variability, loneliness, mindfulness, university students

## Abstract

**Introduction:**

Academic stress represents a multifactorial psychophysiological phenomenon that can disrupt emotional well-being and autonomic balance in university students. Mindfulness-based interventions (MBIs) have shown promise in enhancing emotional regulation and physiological balance. This study aimed to evaluate the autonomic and psychophysiological effects of a 13-week mindfulness-based intervention (MBI) in university students.

**Methods:**

A quasi-experimental longitudinal study was conducted with 93 Colombian university students (86% female; mean age = 18.8 ± 2.35 years). Participants were assigned to an intervention group (*n* = 31) or a control group (*n* = 62). The intervention consisted of weekly 60-min mindfulness sessions over 13 weeks. Psychological (stress, anxiety, depression, loneliness, personality traits, psychological flexibility), behavioral (sleep, physical activity), and autonomic (heart rate and heart rate variability) measures were assessed pre- and post-intervention. Data were analyzed using ANCOVA adjusted for baseline values and relevant covariates (sex, age, sleep), Wilcoxon tests for within-group changes, and effect sizes estimated with Cliff’s delta (*δ*) and rank-biserial correlations (rb). False discovery rate (FDR) correction was applied to control for multiple testing.

**Results:**

After adjusting for covariates, no between-group effects remained significant after FDR correction, although small beneficial trends were observed in conscientiousness and HRV indices. Within-group analyses showed that participants in the intervention group exhibited significant increases in conscientiousness (*p* = 0.039, rb = 0.46), psychological flexibility (*p* < 0.01, rb = −0.51), and reduced loneliness (*p* = 0.006, rb = −0.53). Anxiety and depressive symptoms remained stable in this group but increased in controls, indicating a potential protective effect. No significant changes were observed in perceived stress, sleep, physical activity, or academic performance.

**Conclusion:**

The 13-week mindfulness intervention yielded selective improvements in emotional and autonomic regulation but limited effects on broader psychological and academic outcomes. These findings support a psychobiological model linking mindfulness to enhanced self-regulation and adaptive autonomic modulation. Mindfulness programs may benefit emotional resilience in university settings, though complementary strategies are recommended to enhance broader effects.

**Clinical trial registration:**

https://anzctr.org.au/Trial/Registration/TrialReview.aspx?id=389898, ACTRN12625000984493.

## 1 Introduction

Stress is a multifaceted psychophysiological experience comprising four primary components: the stimulus, the stressor, the immediate physiological response, and the prolonged effects, all of which can vary based on the intensity and nature of the stimulus. At moderate levels, stress can be adaptive, aiding performance in facing challenges. However, when stress becomes excessive or distressing, it activates the central nervous system and disrupts the hypothalamic–pituitary–adrenal (HPA) axis, which governs neuroendocrine hormones. Prolonged activation of this axis may lead to cardiovascular, endocrine, and metabolic dysfunction, along with negative impacts on sleep, cognition, mood, and academic performance (Al-Rouq et al., 2022). Among students, these disruptions are frequently linked to academic overload, poor time management, financial pressures, and uncertainty about the future (Deckard et al., 2022; Loder et al., 2024).

University students encounter a multitude of academic and personal demands daily, often resulting in elevated levels of academic stress. This issue has become increasingly relevant in recent years due to its significant impact on the mental health and well-being of this population (Luceño-Moreno et al., 2023). The global prevalence of academic stress is noteworthy, with reported rates of 22.9% among Chinese students, 68.3% among female medical students in Mexico, and 37.8% in Colombia (Zapata-lópez et al., 2024). In the institution where this study was conducted, students particularly women expressed high levels of perceived stress. Academic stress is linked to symptoms such as depression, anxiety, irritability, and sleep disturbances, especially during exam periods. This often leads to a decline in academic performance due to the demanding nature of the educational environment (Luceño-Moreno et al., 2023; Elamin et al., 2024; Yun and Greenwood, 2022).

To tackle this issue, a range of interventions aimed at stress reduction has been developed, including educational strategies, environmental modifications, gratitude practices, cognitive-behavioral therapies, music therapy, physical activity, exposure to natural light, dietary changes, and animal-assisted therapies (Panahi et al., 2022; Meyer et al., 2024; Aker et al., 2023; De Witte et al., 2022; Li et al., 2023; Syrnyk et al., 2024). Among these approaches, mindfulness has garnered particular attention due to its effectiveness in enhancing emotional and physical well-being, especially in reducing stress, anxiety, and depression. Research indicates that mindfulness practice is especially beneficial in academic settings, where it helps alleviate stress during exam periods and enhances overall academic performance (Sampl et al., 2017; Vorontsova-Wenger et al., 2022; Bennett and Dorjee, 2016). This non-invasive intervention encourages intentional mindfulness training, emphasizing focusing on the present moment without judgment (Bajestani et al., 2024).

A growing body of research has evaluated the effects of mindfulness-based interventions (MBIs) in university populations, showing consistent benefits across psychological, emotional, and academic domains. Recent meta-analyses report that MBIs produce small-to-moderate reductions in perceived stress, anxiety, and depressive symptoms in undergraduate students, with effects typically ranging between g = 0.20 and 0.45 (Dawson et al., 2020; Breedvelt et al., 2019). Improvements in psychological flexibility and emotion regulation have also been observed, suggesting that mindfulness enhances adaptive coping in demanding academic environments (Galante et al., 2018). Studies conducted during periods of high academic pressure, such as exam weeks, also indicate that mindfulness practice can, in some cases, dampen autonomic reactivity and contribute to a moderate increase in heart rate variability, although results in this regard are variable (de Sousa et al., 2021; Kirk and Axelsen, 2020; Hooi et al., 2025). Findings on sleep, physical activity, and academic performance remain inconsistent, with some trials reporting benefits and others showing null effects (Bamber and Kraenzle Schneider, 2016; McConville et al., 2017). Additionally, most previous interventions have been brief (4–8 weeks), relied on self-guided digital formats, or lacked physiological measures, limiting the understanding of linked psychobiological pathways. Therefore, there is a need for studies employing longer programs with combined psychological and autonomic outcomes, particularly using structured, in-person mindfulness sessions as implemented in the present study.

To avoid conceptual ambiguity, it is important to distinguish between dispositional (trait) mindfulness and state mindfulness. Dispositional or trait mindfulness refers to a relatively stable tendency to maintain awareness and attention to present-moment experience in daily life, reflecting an enduring psychological characteristic (Tomlinson et al., 2018). In contrast, state mindfulness represents a momentary condition that fluctuates in response to contextual cues and can be intentionally cultivated during formal mindfulness exercises (Birnkraut et al., 2025). Mindfulness-based interventions primarily target state mindfulness through repeated practice, with the expectation that sustained engagement over time may strengthen trait mindfulness and related self-regulatory capacities (Eberth and Sedlmeier, 2012). In this study, the 13-week program was designed to enhance state mindfulness during weekly sessions, with the potential for downstream effects on trait-like outcomes such as stress perception, emotional regulation, and autonomic balance.

Within a psychobiological framework, mindfulness is conceptualized as a process of attentional training and emotional regulation that influences both subjective experience and physiological functioning. Through sustained attention to the present moment and a non-judgmental awareness of internal states, mindfulness practice is proposed to modulate activity in neural circuits related to stress and self-regulation (including the prefrontal cortex, amygdala, and autonomic centers) (Doll et al., 2016), thereby facilitating adaptive balance between sympathetic and parasympathetic systems. According to this model, improvements in self-awareness and emotion regulation may lead to reductions in perceived stress and loneliness, enhanced conscientiousness, and greater psychological flexibility, which in turn can influence autonomic rhythms such as heart rate and heart rate variability (HRV) (Brown et al., 2021). Thus, the expected causal pathway assumes that mindfulness promotes top–down regulatory control, translating into both psychological benefits (reduced stress, improved emotion regulation) and physiological modulation (more adaptive autonomic variability) (Vargas-Uricoechea et al., 2024). In the present study, these interrelated mechanisms were explored through a set of primary psychological outcomes (e.g., stress, loneliness, conscientiousness) and secondary physiological indicators (HR and HRV), in order to examine the coherence between subjective and autonomic responses to the intervention.

The primary objective of this study was to examine the autonomic and psychophysiological effects of a 13-week mindfulness-based intervention in university students. A secondary objective was to explore whether the intervention was associated with changes in academic performance. The variables analyzed included stress, anxiety, depressive symptoms, personality traits, physical activity, heart rate, academic performance, sleep quality, psychological flexibility, and feelings of loneliness. It was hypothesized that students in the experimental group who participated in the intervention would demonstrate lower levels of perceived stress, anxiety, depressive symptoms, and loneliness compared to the control group, which did not receive the intervention. Additionally, the students in the experimental group would exhibit higher levels of psychological flexibility, improved sleep quality, increased physical activity, and enhanced academic performance following the intervention.

## 2 Methods

### 2.1 Participants

The present study involved 93 Colombian university student volunteers, aged between 16 and 33 years (M = 18.8 ± 2.35), 86% of whom identified as female. Participants were recruited using a non-probability purposive sampling strategy, inviting students who met the inclusion criteria to voluntarily participate. After recruitment, participants were allocated to groups using a quasi-experimental matching procedure, without randomization. Students who enrolled in the mindfulness program comprised the intervention group (*n* = 31; mean age = 18.7 ± 2.86), while the control group (*n* = 62; mean age = 18.8 ± 2.08) was formed by selecting two matched participants for each member of the intervention group following a 2:1 ratio. Matching was based on age and weight, while other variables (e.g., sex, academic major, socioeconomic status, or motivation toward mindfulness) were not controlled in the allocation process.

Participants completed an online questionnaire at two time points: at the beginning of the academic semester (February 2024) and at the end of the academic period (May 2024). Inclusion criteria required participants to be enrolled in the current academic year, reside in Colombia, be a student in any academic program, and not have diagnosed medical conditions or be taking medication. To prevent duplicate responses, students provided their student ID, which was verified against the university database. All collected information was treated confidentially.

The study protocol was reviewed and approved *prior to data collection* by the institutional ethics committees of both the Universidad Europea de Madrid (Spain), where the research project is formally registered, and the Colombian university where data collection was conducted [Ethics Committee approval code: CIPI/2024(611)]. All procedures were conducted in accordance with the Declaration of Helsinki and Colombian regulations for research involving human participants (Resolution 8,430 of 1993). All participants provided digitally signed informed consent before participation. For participants under 18 years of age, informed assent was obtained from the student, together with written informed consent from a parent or legal guardian, in accordance with institutional and national ethical guidelines.

The study was retrospectively registered with the Australian New Zealand Clinical Trials Registry (ANZCTR; ACTRN12625000984493) during the publication process, as the original study design was not classified as a clinical trial at the time of implementation. This retrospective registration does not imply *post hoc* modifications to the study protocol, outcomes, or analysis plan.

### 2.2 Procedure

To achieve the objective of this research, a longitudinal study was conducted with two measurements taken over time: one before the start of the mindfulness-based intervention and another at the end of the academic period, thirteen weeks later. The instruments were administered on the university campus before classes began. Student academic performance was measured on a scale of 1 to 5, where 1 is low academic performance and 5 is high academic performance. A reduced version of the Spanish adaptation of the Big Five Inventory was used to assess personality traits such as openness to experience, conscientiousness, extraversion, agreeableness, and neuroticism. This abbreviated version consists of 10 items rated on a 5-point Likert scale, where 1 indicates strong disagreement and 5 indicates strong agreement (Rammstedt and John, 2007). The scale showed good reliability, achieving a Cronbach’s *α* of 0.70, except for the openness to experience factor, which had a value of 0.65 (Robles-Haydar et al., 2022). Additionally, the Spanish version of the Zung Depression Scale was used to assess depression levels (Zung, 1965); this scale demonstrated strong reliability, with a Cronbach’s α of 0.85 (Del Águila Montoya et al., 2021). The Spanish version of the UCLA Loneliness Scale was used to measure loneliness (Russell, 1996). This scale assesses loneliness. A condensed version with three items, rated on a three-point Likert scale where 1 represents “never” and 3 represents “frequently,” was utilized in this study. The reliability of this test varied between 0.89 and 0.94 (Russell, 1996). A reduced version of the Spanish adaptation of the Spielberger State–Trait Anxiety Inventory (Van Knippenberg et al., 1990), consisting of 6 items that assess anxiety and are rated on a 4-point Likert scale, where 1 indicates “not at all” and 4 indicates “very much,” was used to measure anxiety. The reliability of this test ranges from 0.85 to 0.93 (Castellote-Caballero et al., 2024; González-Álvarez et al., 2025). The Spanish version of the Acceptance and Action Questionnaire II (Bond et al., 2011) was employed to assess experiential avoidance or psychological inflexibility through 7 items rated on a 7-point Likert scale, where 0 represents “never true” and 7 represents “always true”. The reliability of this test is measured at 0.84 (Bond et al., 2011). The PSS-4, adapted by Herrero and Meneses (2006), was used to measure perceived stress. This 4-item scale evaluates how often individuals feel stressed, with higher scores indicating greater perceived stress. Although Herrero and Meneses used a 1 to 5 scale, the original 0 to 4 scale by Cohen was applied in this study, where 0 represents “never” and 4 represents “very often.” The scale demonstrated good reliability, with a Cronbach’s *α* of 0.72, and accounted for 54% of the variance (Herrero and Meneses, 2006).

To minimize participant fatigue and ensure feasibility within the classroom-based assessments, abbreviated and validated versions of all questionnaires were used. The decision to employ brief versions was based on prior evidence supporting their construct validity and sensitivity to change in non-clinical populations. The use of the original 0–4 Likert format for the PSS-4 ensured consistency with the original validation studies by Cohen et al. (1983) and comparability with other mindfulness-based intervention research. Internal consistency coefficients (Cronbach’s *α*) in the present sample ranged from 0.72 (PSS-4) to 0.88 across instruments, confirming adequate reliability for research purposes. These evaluations were carried out in the first period of the academic semester. Although most instruments used in this study were originally validated in Spanish populations from Spain or Latin America, their linguistic equivalence and psychometric robustness support their use in Colombian university samples. To ensure their adequacy for the present context, a brief pilot test was conducted with a small group of students to assess item clarity and cultural appropriateness. No difficulties in comprehension were reported, and internal consistency coefficients obtained in the current sample ranged from acceptable to high (α = 0.72–0.88), supporting the reliability of these measures within the Colombian university population.

Behavioral patterns of participants were evaluated in line with previous studies (Peris-Ramos et al., 2024; Jiménez-Morcillo et al., 2024; Martín-Rodríguez et al., 2022). Hours of sleep per day were measured on a self-perception scale, indicating the number of hours the student sleep per day. The quality of the percipients’ last sleep was measured using a Likert scale, where 1 means very poor sleep quality and 10 means very good sleep quality. The average number of steps per day in the last week was measured on a self-perception scale, indicating the number of steps the student had taken in the last week. Physical activity habits were measured with a questionnaire used in line with previous research. The psychophysiological response to stress in contexts of high psychological demand was assessed by means of a questionnaire including the items: “Did you do any physical activity in the last 7 days?,” “If so, time in minutes of cyclic and/or aerobic activity (cycling, treadmill, Zumba) adding up all the sessions of the 7 days,” “If so, time in minutes of activity with self-loads (sit-ups, push-ups, squats) or weights (gym machines, weights) adding up all the sessions of the 7 days.” Although these measures relied on self-report and single-item indicators rather than validated multi-item instruments, they were included as exploratory outcomes to provide an initial overview of behavioral patterns associated with stress and mindfulness practice. This approach was adopted to minimize participant burden and accommodate the classroom-based data collection schedule.

The autonomic modulation was assessed through the analysis of heart rate variability (HRV). The HRV data were collected using the EEG for Everybody mobile device (NoviSad, Serbia) following previous procedures (Regueros et al., 2023). This device incorporates a validated single-lead ECG sensor that provides HRV metrics comparable to those obtained with standard ECG systems in controlled settings. Recordings were conducted in accordance with current HRV measurement guidelines (Cardiology, 1996; Forte et al., 2019). All measurements were carried out between 9:00 a.m. and 12:00 p.m. to control for circadian variations. Participants were seated comfortably in an upright position with feet flat on the floor and hands resting on their thighs, in a quiet room maintained at 22–24 °C. They were instructed to abstain from caffeine, exercise, and heavy meals for at least 2 hours prior to testing. Following a 10-min adaptation period, HRV was recorded for 5 min during spontaneous breathing at rest. Real-time inspection of the signal was performed to minimize artifacts, and segments with more than 5% artifact contamination were excluded and repeated. The variables analyzed included heart rate (HR), rMSSD (square root of the mean squared differences between adjacent normal R–R intervals), pNN50 (percentage of differences between adjacent normal R–R intervals greater than 50 ms), standard deviation 1 (SD1), standard deviation 2 (SD2), the ratio between SD1 and SD2 (SD1/SD2), low frequency (LF), high frequency (HF), the ratio of low to high frequency (LF/HF), low frequency in normalized units (LFnu), and high frequency in normalized units (HFnu). This protocol has been successfully implemented in prior psychophysiological research with young adult and university samples, showing stable signal quality and consistent HRV responses to stress-related paradigms (Benítez-Agudelo et al., 2025a, 2025b).

After the first measurement of the participants, we initiated a Mindfulness-based intervention aimed at alleviating academic stress among students. This program spanned 13 weeks, with sessions held for 1 h each Thursday in a quiet, enclosed space, free from distractions and scheduled before the start of classes. The Mindfulness sessions were organized as detailed in Table 1. The program was facilitated by a licensed psychologist trained in Mindfulness-Based Stress Reduction (MBSR), with experience conducting mindfulness-based interventions in educational and clinical contexts. Each session followed a structured thematic manual including guided practices (e.g., body scan, mindful breathing, awareness of emotions), reflective discussions, and brief psychoeducational components. Participants were encouraged to engage in informal daily mindfulness practices for approximately 10 min between sessions, such as mindful breathing or attention to bodily sensations. Attendance was recorded weekly, and participants reported the frequency and perceived usefulness of their personal practice at the end of the intervention. To ensure fidelity, the facilitator adhered to a standardized session plan and thematic sequence throughout the program, maintaining consistency in the delivery and timing of exercises. Although no external fidelity checklist was implemented, supervision meetings were periodically held to review adherence to the intervention protocol. At the conclusion of the 13 weeks, a final measurement of the study variables was conducted.

**Table 1:** Mindfulness sessions.

Week	Objective	Development	Material
Week 1 - Introduction to mindfulness	Introduce students to the concept of mindfulness and its benefits for stress management.	The session begins with a presentation of what mindfulness is, space is opened to solve doubts and discuss, and basic conscious breathing exercises are performed.	Slides, guided meditation audios.
Week 2 - Body awareness	Teach students to be aware of bodily sensations and use them to anchor attention.	The session begins with a body scan exercise and ends with a group discussion about what was experienced during the session.	Body scan audios, mats.
Week 3 - Mindfulness in breathing	Deepen the practice of mindfulness in the breath.	The session begins with a practice of conscious breathing and techniques to manage distractions are taught and practiced.	Printed breathing guides.
Week 4 - Mindfulness in daily life	Integrate mindfulness practices into everyday activities.	The session begins with mindfulness exercises during activities such as walking or eating, then a discussion is made on how to incorporate mindfulness in the study and ends with a guided meditation session.	Practice guides, mindfulness journals.
Week 5 - Stress management through mindfulness	Apply mindfulness to manage stressful situations.	The session begins with breathing techniques for times of stress, followed by a role-playing of stressful situations and mindfulness response practice.	Written scenarios, breathing technique guides.
Week 6 - Mindfulness and emotions	Learn to observe and manage emotions through mindfulness.	The session begins with a guided meditation focused on emotional awareness and ends with a discussion on the relationship between emotions, thoughts, and behavior.	Meditation audios, worksheets for reflection. (Make a drawing that expresses the relationship between emotion, thought and behavior).
Week 7 - Building resilience	Strengthen resilience through mindfulness practices.	The session begins with positive visualization exercises (as we imagine ourselves in our ideal self) a message of gratitude is given to oneself; It ends with a discussion on how to face challenges with a resilient mindset.	Viewing audios, gratitude journals.
Week 8 - Concentration and mental clarity	Improve concentration and mental clarity through mindfulness.	The session begins with a meditation practice focused on concentration, ending by teaching techniques to improve mental clarity during study.	Meditation audios, mindful study guides.
Week 9 - Mindful communication	Apply mindfulness in daily communication.	The session begins with active listening and conscious communication exercises and ends with a role-playing of communication situations.	Role-playing scenarios, communication guides.
Week 10 - Mindfulness and Self-compassion	Cultivate self-compassion through mindfulness practices.	The session begins with a guided meditation on self-compassion, ending with exercises to develop a positive internal dialogue.	Meditation audios, worksheets.
Week 11 - Test preparation with mindfulness	Use mindfulness techniques to prepare for exams.	The session begins with specific mindfulness strategies for study and exam preparation (Discussion of what we can apply to mindfulness for exams), the session ends by creating a mindful study plan.	Curricula, practice guides.
Week 12 - Integration of knowledge	Review and consolidate the mindfulness skills learned.	The session begins by giving a space for the questions that have arisen during the weeks of the intervention and closes with a meditation and reflection on the process.	Journals of reflection.
Week 13 - Closing and consolidation	Reflect on what you have learned and how you can continue to apply it	The session begins with guided breathing exercises, then a painting session on canvas is carried out and ends with a meditation.	Canvases, paintings, certificates of participation.

### 2.3 Statistical analysis

Statistical analyses were performed using IBM SPSS Statistics version 24.0 (SPSS Inc., Chicago, IL, USA) and R version 4.3. Descriptive statistics (mean and standard deviation) were computed for all variables. The Kolmogorov–Smirnov test indicated that the data did not meet the assumption of normality; therefore, nonparametric and robust approaches were employed. Between-group comparisons at posttest were performed using analysis of covariance (ANCOVA) models, with post-intervention scores as dependent variables, group (intervention vs. control) as a fixed factor, and baseline (pretest) values as covariates. Sex, age, and baseline sleep hours were also included as additional covariates to control for potential confounding effects. From each model, adjusted mean differences, 95% confidence intervals, and F-statistics were obtained. To estimate nonparametric effect sizes for between-group contrasts, Cliff’s delta (*δ*) was computed along with 95% bootstrap confidence intervals (2,000 resamples). Given the number of simultaneous tests, the Benjamini–Hochberg false discovery rate (FDR) correction was applied to control for type I error. Within-group changes from pretest to posttest were analyzed using the Wilcoxon signed-rank test, and the corresponding effect sizes were expressed as the rank-biserial correlation (rb) with 95% bootstrap confidence intervals. Mean pre–post differences (*Δ* = post − pre) and their standard deviations were reported for descriptive purposes. Effect size magnitudes were interpreted following conventional criteria: for Cliff’s *δ*, |δ| < 0.147 = negligible, 0.147–0.33 = small, 0.33–0.474 = medium, and ≥ 0.474 = large; for rank-biserial correlations, |rb| < 0.10 = trivial, 0.10–0.30 = small, 0.30–0.50 = moderate, and ≥ 0.50 = large. The statistical significance level was set at *p* ≤ 0.05.

## 3 Results

The results presented in Table 2 show the adjusted between-group comparisons after the 13-week mindfulness intervention, controlling for baseline scores, sex, age, and baseline sleep duration. The analyses were conducted using analysis of covariance (ANCOVA) and complemented with nonparametric effect sizes (Cliff’s *δ*) and *p*-value correction for multiple comparisons (Benjamini–Hochberg FDR).

**Table 2:** Adjusted effects of the 13-week mindfulness intervention.

Variable		Adj. mean diff (95% CI)	F	*p*	pFDR	Cliff’s δ (95% CI)	Interpretation
Biomedical	Quality of sleep	0.10 (−0.81–1.01)	0.05	0.83	0.94	−0.01 (−0.26–0.23)	No significant effect
Personality	BIG-FIVE Extraversion	0.14 (−0.48–0.77)	0.21	0.65	0.92	0.10 (−0.15–0.37)	No significant effect
	BIG-FIVE Agreeableness	−0.08 (−0.62–0.46)	0.09	0.77	0.94	−0.11 (−0.34–0.13)	No significant effect
	BIG-FIVE Conscientiousness	0.84 (0.18–1.49)	6.49	0.013	0.26	0.25 (0.00–0.47)	Small–moderate ↑ (ns after FDR)
	BIG-FIVE Neuroticism	−0.31 (−0.95–0.34)	0.89	0.35	0.78	−0.12 (−0.36–0.10)	No effect
	BIG-FIVE Open to Experience	−0.24 (−0.91–0.43)	0.51	0.48	0.86	−0.23 (−0.46–0.00)	No effect
Anxiety	STAI	−0.93 (−2.72–0.87)	1.05	0.31	0.78	0.03 (−0.22–0.26)	No effect
Psychological flexibility	AAQII	0.28 (−3.66–4.22)	0.02	0.89	0.94	0.12 (−0.12–0.35)	No effect
Solitude	UCLA	−0.22 (−0.96–0.52)	0.35	0.56	0.86	0.10 (−0.14–0.35)	No effect
Perceived stress	PSS-4	0.02 (−1.21–1.25)	0.00	0.98	0.98	−0.03 (−0.27–0.21)	No effect
Symptoms of depression	ZUNG Scale	0.31 (−3.20–3.82)	0.03	0.86	0.94	0.21 (−0.05–0.45)	No effect
Physical activity	Steps	2205.20 (−5411.27–9821.66)	0.35	0.56	0.86	−0.30 (−0.62–0.06)	No effect
	Cyclical/aerobic activity	−0.10 (−0.44–0.24)	0.20	0.65	0.91	−0.09 (−0.34–0.17)	No effect
	Weight/abs activity	−0.15 (−0.56–0.26)	0.53	0.47	0.86	−0.07 (−0.33–0.18)	No effect
Heart rate variability	HR	−3.15 (−10.46–4.17)	0.73	0.40	0.79	0.07 (−0.19–0.32)	No effect
	rMSSD	−7.87 (−16.66–0.92)	3.17	0.079	0.40	−0.18 (−0.41–0.09)	Trend to decrease (ns)
	PNN50	−5.20 (−12.92–2.52)	1.79	0.184	0.70	−0.15 (−0.39–0.10)	No effect
	SD1	−3.19 (−9.92–3.53)	0.89	0.35	0.78	−0.16 (−0.40–0.10)	No effect
	SD2	−8.81 (−18.39–0.78)	3.34	0.071	0.40	−0.21 (−0.44–0.04)	Trend to decrease (ns)
	Lfnu	5.52 (−3.15–14.19)	1.60	0.209	0.70	0.13 (−0.14–0.39)	No effect
	Hfnu	−8.16 (−17.06–0.74)	3.32	0.072	0.40	−0.22 (−0.46–0.04)	Trend to decrease (ns)
Academic performance	Evaluation grade	0.03 (−0.28–0.34)	0.03	0.87	0.94	−0.04 (−0.26–0.21)	No effect

Adj. mean diff: adjusted mean difference between groups (Intervention – Control) estimated using analysis of covariance (ANCOVA), controlling for baseline score, sex, age, and baseline sleep duration (hours of sleep per day). CI: 95% confidence interval. F and *p* values correspond to the group effect in the ANCOVA model. pFDR = *p*-value corrected for multiple comparisons using the Benjamini–Hochberg false discovery rate (FDR) procedure. Cliff’s δ: nonparametric effect size for between-group differences at posttest; values of |δ| ≈ 0.11, 0.28, and 0.43 represent small, medium, and large effects, respectively. STAI, State–Trait Anxiety Inventory; AAQII, Acceptance and Action Questionnaire II; PSS-4, Perceived Stress Scale (4 items); HR, Heart rate; rMSSD, Root mean square of successive differences between normal RR intervals; PNN50, Percentage of successive RR intervals differing by more than 50 ms; SD1, Short-term HRV component; SD2, Long-term HRV component; LFnu, Low-frequency normalized units; HFnu, High-frequency normalized units. All variables were analyzed using the same model structure: post ~ group + pre + sex + sleep_pre.

Regarding biomedical variables, no significant adjusted differences were found between the intervention and control groups in sleep quality (*p* = 0.83, *pFDR* = 0.94; *δ* = −0.01, 95% CI [−0.26, 0.23]), suggesting that the intervention did not influence subjective sleep quality when baseline differences were controlled.

In the personality dimensions assessed by the BIG-FIVE, only Conscientiousness (Scrupulous) showed a small-to-moderate positive adjusted difference favoring the intervention group (*F* (1.88) = 6.49, *p* = 0.013, *pFDR* = 0.26, *δ* = 0.25), although this effect did not remain significant after FDR correction. The remaining traits (Extraversion, Agreeableness, Neuroticism, and Openness to Experience) did not exhibit significant adjusted differences (all *pFDR* > 0.70).

For anxiety (STAI) and psychological flexibility (AAQII), no significant adjusted effects were observed (*pFDR* = 0.78 and 0.94, respectively), indicating that the mindfulness intervention did not produce measurable changes in these constructs beyond baseline differences. Similarly, loneliness (UCLA), perceived stress (PSS-4), and depressive symptoms (ZUNG Scale) showed no significant differences between groups (*pFDR* > 0.80), although the direction of effects suggested slight improvements in the intervention group (Cliff’s *δ* ranging from 0.10 to 0.21).

With respect to physical activity, adjusted group differences in steps, aerobic activity, and strength exercises were not statistically significant (*pFDR* = 0.86–0.91), and all Cliff’s δ values were below 0.30, indicating minimal effects of the intervention on objective activity metrics.

In the autonomic regulation domain, small, non-significant adjusted differences were observed in rMSSD (*p* = 0.079, *pFDR* = 0.40, δ = −0.18), SD2 (*p* = 0.071, *pFDR* = 0.40, δ = −0.21), and HFnu (*p* = 0.072, *pFDR* = 0.40, δ = −0.22), all trending toward decreased variability in the intervention group. Although these effects did not reach statistical significance, they may indicate subtle changes in autonomic modulation associated with mindfulness practice.

Finally, academic performance (Evaluation grade) did not differ between groups (*pFDR* = 0.94, δ = −0.04), suggesting that the intervention did not directly influence academic achievement during the study period.

Overall, after adjusting for covariates and controlling the false discovery rate, no variable reached statistical significance. Nevertheless, the pattern of results indicates small beneficial trends in conscientiousness and autonomic indices, consistent with partial improvements in self-regulation and physiological balance associated with mindfulness practice.

Table 3 summarizes the within-group pre–post changes in the study variables for both the control and intervention groups, using the Wilcoxon signed-rank test and rank-biserial correlation (rb) as a nonparametric effect size indicator. Overall, the results show that few variables exhibited significant within-group changes, indicating that most measures remained relatively stable across time, particularly in the control condition.

**Table 3:** Within-group pre–post changes (Wilcoxon test and effect sizes).

Variable		Δ Control (M ± SD)	Wilcoxon *p*	rb (95% CI)	Δ Intervention (M ± SD)	Wilcoxon *p*	rb (95% CI)
Biomedical	Quality of sleep	−0.52 ± 2.21	0.094	−0.24 (−0.51–0.02)	−0.29 ± 2.90	0.541	−0.13 (−0.54–0.28)
Personality	BIG-FIVE Extraversion	0.03 ± 1.77	0.793	0.04 (−0.22–0.32)	0.19 ± 1.25	0.606	0.14 (−0.40–0.59)
	BIG-FIVE Agreeableness	0.07 ± 1.36	0.679	0.06 (−0.23–0.34)	0.10 ± 1.33	0.610	0.12 (−0.34–0.62)
	BIG-FIVE Conscientiousness	−0.36 ± 1.43	0.063	−0.26 (−0.49–0.01)	0.71 ± 1.79	0.039	0.46 (0.06–0.74)
	BIG-FIVE Neuroticism	0.18 ± 1.64	0.321	0.14 (−0.14–0.40)	−0.16 ± 1.86	0.566	−0.14 (−0.60–0.33)
	BIG-FIVE Openness to Experience	−0.24 ± 1.69	0.336	−0.14 (−0.41–0.15)	−0.13 ± 1.38	0.854	0.04 (−0.40–0.53)
Anxiety	STAI	1.32 ± 4.00	0.019	0.32 (0.06–0.54)	−0.42 ± 5.12	0.630	−0.09 (−0.44–0.26)
Psychological flexibility	AAQ-II	−2.84 ± 9.96	0.003	−0.39 (−0.60−−0.14)	−3.94 ± 7.16	0.004	−0.51 (−0.77−−0.20)
Solitude	UCLA Loneliness	−0.24 ± 1.89	0.259	−0.17 (−0.44–0.13)	−0.94 ± 1.69	0.006	−0.53 (−0.79−−0.19)
Perceived stress	PSS-4	0.52 ± 3.52	0.532	0.08 (−0.18–0.34)	0.94 ± 3.63	0.160	0.26 (−0.13–0.59)
Symptoms of depression	ZUNG Depression Scale	2.87 ± 7.40	0.004	0.37 (0.14–0.60)	0.32 ± 8.63	0.779	0.06 (−0.31–0.39)
Physical activity	Steps	−1240.14 ± 11187.0	1.000	0.00 (−0.47–0.43)	1113.16 ± 8821.2	0.657	0.13 (−0.48–0.70)
	Cyclical/aerobic activity	−39.1 ± 274.6	0.889	−0.05 (−0.89–0.64)	23.6 ± 63.9	0.461	0.30 (−0.47–0.90)
	Weight/abs activity	−137.8 ± 248.8	0.161	−0.50 (−0.84–0.15)	−174.0 ± 234.7	0.144	−0.73 (−0.91–0.18)
Heart rate variability (HRV)	HR	2.19 ± 15.41	0.325	0.13 (−0.13–0.36)	−4.77 ± 21.25	0.100	−0.30 (−0.62–0.05)
	rMSSD	−1.53 ± 29.08	0.749	−0.04 (−0.30–0.22)	−7.10 ± 19.72	0.028	−0.40 (−0.68−−0.07)
	PNN50	−4.97 ± 26.92	0.289	−0.14 (−0.38–0.11)	−4.03 ± 17.85	0.247	−0.21 (−0.53–0.15)
	SD1	−1.60 ± 21.24	0.664	−0.06 (−0.31–0.21)	−5.32 ± 14.32	0.024	−0.25 (−0.46–0.04)
	SD2	1.22 ± 28.41	0.822	0.08 (−0.16–0.32)	−4.13 ± 31.57	0.357	−0.18 (−0.41–0.09)
	LFnu	−0.58 ± 28.90	0.616	−0.06 (−0.31–0.21)	−1.64 ± 18.05	0.710	−0.07 (−0.42–0.28)
	HFnu	5.35 ± 30.04	0.246	0.17 (−0.08–0.41)	0.89 ± 17.12	0.754	−0.06 (−0.43–0.29)
Academic performance	Evaluation grade	0.10 ± 0.82	0.393	0.11 (−0.15–0.34)	0.05 ± 0.69	0.066	0.33 (−0.03–0.69)

Δ = Post − Pre; Wilcoxon p = two-tailed Wilcoxon signed-rank test within each group; rb = rank-biserial correlation (effect size for paired data); 95% CI estimated with 2000 bootstrap replications. STAI, State–Trait Anxiety Inventory; AAQ-II, Acceptance and Action Questionnaire II; PSS-4, Perceived Stress Scale; HR, Heart rate; rMSSD, root mean square of successive differences; PNN50, % of RR intervals > 50 ms; SD1, short-term HRV; SD2, long-term HRV; LFnu/HFnu, normalized low/high frequency components; M, mean; SD, standard deviation.

In the personality dimensions, a significant increase in conscientiousness was observed in the intervention group (*p* = 0.039, rb = 0.46), suggesting that mindfulness training may have promoted greater responsibility and self-regulation compared to baseline levels. The control group, in contrast, showed a nonsignificant trend toward a decrease in this trait (*p* = 0.063, rb = −0.26). The other Big Five traits (extraversion, agreeableness, neuroticism, and openness to experience) did not exhibit statistically significant pre–post changes in either group.

Regarding psychological and emotional variables, anxiety levels (STAI) significantly increased in the control group (*p* = 0.019, rb = 0.32), whereas no significant change was detected in the intervention group, suggesting a potential buffering effect of mindfulness practice against stress and anxiety. Psychological inflexibility (AAQ-II) significantly decreased in both groups (*p* < 0.01), with a larger effect in the intervention group (rb = −0.51), indicating improved psychological flexibility after the intervention. Moreover, perceived loneliness (UCLA) showed a significant reduction in the intervention group (*p* = 0.006, rb = −0.53), but not in the control group, suggesting that mindfulness training may have enhanced social connectedness and reduced feelings of isolation.

For the biomedical and mood-related measures, depressive symptoms (ZUNG) significantly increased in the control group (*p* = 0.004, rb = 0.37), while remaining stable in the intervention group, reinforcing the protective role of the intervention on emotional well-being. No significant changes were found in sleep quality or perceived stress (PSS-4) in either group.

Concerning physiological regulation, several heart rate variability (HRV) indicators showed trends in the expected direction among participants receiving the intervention. Specifically, heart rate (HR) exhibited a nonsignificant trend toward reduction (*p* = 0.100, rb = −0.30), while rMSSD and SD1, indicators of parasympathetic modulation, significantly decreased (*p* = 0.028 and *p* = 0.024, respectively), reflecting possible short-term autonomic adjustments to the mindfulness practice. Other HRV metrics (PNN50, SD2, LFnu, HFnu) did not present significant pre–post differences.

Finally, no significant changes were found in physical activity or academic performance in either group, suggesting that the intervention primarily influenced psychological flexibility, conscientiousness, and emotional regulation rather than behavioral outcomes. Overall, these findings indicate that the mindfulness-based intervention was associated with improvements in psychological flexibility, reductions in loneliness, and stabilization of anxiety and depressive symptoms, whereas the control group tended to show slight deteriorations in affective and self-regulatory domains.

## 4 Discussion

This study assessed the autonomic and psychophysiological effects of a 13-week mindfulness-based intervention in university students. The findings offer partial but cautious support for the initial hypotheses. The intervention group exhibited a trend toward greater conscientiousness and reduced heart rate. However, no significant changes were noted in variables such as perceived stress, anxiety, or depressive symptoms when compared to the control group. Additionally, participants in the intervention group showed a tendency toward greater psychological flexibility, reduced perceptions of loneliness, and apparent stabilization in symptoms of anxiety and depression. Nonetheless, a decline in sleep quality was also reported, and no significant changes in academic performance were identified relative to baseline measurements, suggesting a limited impact of the intervention in these areas throughout the study period.

Regarding sleep variables, a decline in both sleep hours and quality was observed in both groups during the final assessment. Given the timing of data collection coinciding with end-of-semester academic demands, this pattern likely reflects contextual stress rather than intervention effects. However, the study did not include a sensitivity analysis by academic calendar, which limits our ability to confirm this interpretation empirically. These findings contrast with other studies that highlight the positive effects of mindfulness on sleep quality (Fu et al., 2022; Xiong et al., 2023; Ling et al., 2024). Nonetheless, some research indicates that for optimal outcomes, mindfulness interventions should be combined with additional strategies, such as cognitive-behavioral therapies, particularly in high-stress academic environments (Blake et al., 2016). Despite this, the beneficial relationship between mindfulness and sleep quality can be linked to mindfulness’s effectiveness in reducing stress and enhancing emotional balance, key factors in regulating sleep (Ye et al., 2022). Importantly, the absence of sleep improvements in this study should not be interpreted as evidence of inefficacy, but rather as a potential ceiling effect of contextual stressors overriding mindfulness benefits. This interpretation aligns with the idea that academic stress acts as a competing demand on cognitive and physiological resources, limiting the observable benefits of contemplative practice during peak workload periods (Chen et al., 2020; Rusch et al., 2019). Future research employing ecological momentary assessment or repeated sleep-tracking could help clarify whether mindfulness buffers sleep deterioration dynamically rather than at discrete timepoints.

In terms of physical activity, no significant changes were observed in any group, probably due to the increased workload at the end of the semester and the reduction in students’ time for other activities. This finding differs from the results of other studies, including those by Ullrich-French et al. (2017) and Murphy et al. (2012), which suggest that mindfulness is associated with increased body awareness and adoption of healthier behaviors, including physical activity. Given that physical activity and sleep were measured with single-item indicators, the precision and sensitivity of these measures may not have been sufficient to detect subtle changes, emphasizing the importance of using validated, multi-item or objective measures in future research.

Following the intervention, we observed a decrease in loneliness among students in the intervention group. This finding suggests that mindfulness may foster social connectedness through shared group experiences and increased self-awareness. This improvement may be attributed to the group dynamics and shared reflections experienced during the sessions. Furthermore, these results are consistent with previous research indicating that mindfulness-based interventions lasting eight weeks or more significantly reduce feelings of loneliness in college students (Rubin et al., 2024; Sun, 2023). This effect may also reflect the interpersonal components of group-based mindfulness sessions, such as emotional disclosure, perceived belonging, and resonance with others’ experiences, which are increasingly recognized as active therapeutic mechanisms in contemplative training (Jong et al., 2025; Silveira et al., 2023). However, this effect was small and should be interpreted as a tentative outcome rather than conclusive evidence, especially considering the modest sample size and the lack of long-term follow-up.

Similarly, the increase in conscientiousness may reflect context-dependent self-regulatory engagement rather than a stable change in trait structure. Given that the personality measure employed an abbreviated two-item-per-trait scale, the observed effect might capture short-term shifts in response style, motivation, or positive affect, rather than a fundamental alteration in the conscientiousness dimension. This interpretation aligns with evidence that mindfulness can temporarily enhance attentional control and behavioral regulation (Karl et al., 2021), but further longitudinal studies are needed to determine whether these effects persist beyond transient states. Recent frameworks conceptualize such short-term trait fluctuations as “state–trait dynamics,” where state mindfulness produces immediate regulatory benefits that may accumulate into trait-level changes with sustained practice (Wu and Ma, 2025). This may help explain the directionality observed despite measurement constraints.

Although no significant between-group differences were observed in state anxiety or perceived stress, participants in the mindfulness-based intervention group maintained stable anxiety levels throughout the semester, whereas the control group tended to experience increased symptoms. This may suggest that participants needed additional time to fully internalize the benefits of mindfulness in stressful situations, or that the intervention itself may not have been sufficiently prolonged or intensive to adequately address the perceived academic pressures. These findings contrast with those reported by Baer et al. (2012), which indicated that shorter, 8-week interventions were effective in significantly reducing perceived stress levels. However, differences in population characteristics, such as the developmental stage of the participants, could affect the intervention’s overall effectiveness. On the other hand, research involving university students has shown reductions in stress and anxiety levels following mindfulness courses, particularly during examination periods, reinforcing the notion that these interventions enhance psychological resilience in the face of academic challenges (Galante et al., 2018). Taken together, these mixed findings highlight mindfulness’s potential to function more reliably as a protective factor preventing symptom exacerbation, rather than as a rapid symptom-reduction tool under high academic pressure (Linardon et al., 2024; Kim et al., 2025).

Conversely, both groups exhibited a reduction in psychological inflexibility, indicating an enhanced capacity to accept challenging internal experiences without allowing them to disrupt value-oriented behavior. This finding underscores the importance of mindfulness in fostering psychological flexibility, particularly when integrated with value-based interventions, which can bolster self-regulation and strengthen alignment with personal goals (Aydin and Aydin, 2021). Previous research supports the efficacy of mindfulness as a practical approach to improving mental flexibility; however, further investigation is needed to explore how values influence this process and augment its effects (Fahimi et al., 2021). Regarding depressive symptoms, while no significant changes were observed in either group overall, a rise in such symptoms was noted within the control group by the semester’s end, suggesting a potential protective effect of mindfulness in the experimental group. This aligns with existing literature that highlights the significant and clinically relevant impact of mindfulness-based interventions in reducing anxiety and depression, with improvements often sustained over time. This reinforces the notion that mindfulness may serve a protective function in regulating depressive symptoms within academic settings, as evidenced by the current study (Khoury et al., 2013; Jiang et al., 2023).

From a physiological standpoint, the observed changes in heart rate (HR) and heart rate variability (HRV) metrics reflect subtle and mixed adaptations in autonomic regulation linked to the intervention. Although the adjusted analyses revealed small, non-significant reductions in HR and decreases in rMSSD, SD2, and HFnu, these findings suggest nuanced modulations rather than unequivocal enhancement of vagal control. The absence of significant parasympathetic increases indicates that mindfulness may have promoted a mild autonomic recalibration without producing a consistent relaxation response. This pattern could represent a partial adaptation to academic stress, where the intervention helped stabilize physiological responses but was insufficient to counteract the cumulative demands of the semester. Supporting this observation, previous studies have demonstrated that mindfulness practice can aid in the recovery of cardiovascular responses to stress. For instance, Koerten et al. (2020) found that incorporating the nonjudgmental component of mindfulness improved cardiovascular recovery in perfectionistic college students. Nevertheless, in high-stress contexts such as university settings, this regulatory effect may be attenuated or emerge gradually over time. Additionally, research by Gomutbutra et al. (2022) revealed that mind–body interventions can lead to positive changes in biomarkers associated with neuroplasticity, such as brain-derived neurotrophic factor (BDNF). This suggests that while mindfulness can enhance both physiological and psychological well-being, its effects on autonomic flexibility may require longer practice durations, lower contextual stress, or integration with complementary self-regulation strategies. Physiologically, this aligns with neurovisceral integration models, which propose that vagal enhancement is more likely to occur after sustained practice once cognitive–emotional regulatory networks consolidate (Ha et al., 2025; Schneider et al., 2025). Thus, the subtle HR and HRV changes observed may represent early autonomic shifts that were not yet strong enough to manifest as significant group differences.

Regarding academic performance, no significant changes in post-intervention were observed, potentially due to factors such as the level of academic demand, the duration of the intervention, and the impact of other uncontrolled variables. While prior studies (Mrazek et al., 2013; Lin and Mai, 2018) have indicated that mindfulness courses implemented prior to exams can enhance academic performance, our findings may underscore the complexity of this relationship. According to Yerkes and Dodson’s inverted U model, moderate levels of activation are conducive to optimal performance, whereas extremely low or high levels can be detrimental. Despite the lack of overall improvements, it is plausible that mindfulness may have assisted some students in better managing stress and reducing the risk of academic dropout. This highlights the need for future studies to explore these effects in greater depth (Galante et al., 2018). The absence of performance changes in this study reinforces the idea that academic outcomes may require either more intensive training or targeted cognitive components (e.g., attentional monitoring, metacognitive awareness) to translate into measurable improvement (Nwadi et al., 2025; Charness et al., 2024).

In terms of limitations, several factors should be acknowledged. First, the imbalance in group sizes, reliance on self-reported questionnaires, and the lack of continuous monitoring of physiological biomarkers may have influenced the findings. Moreover, the quasi-experimental design with non-random allocation represents a methodological constraint that may have introduced selection bias and residual confounding. Although participants were matched by age and weight and both groups were assessed within the same academic semester, other relevant variables such as sex, socioeconomic status, academic major, and baseline motivation toward mindfulness were not controlled and could have affected the results. Additionally, the measures of physical activity and sleep were based on brief self-reported, single-item indicators, which may limit accuracy and increase unexplained variance. These variables were included as exploratory outcomes due to the classroom-based and time-constrained context of data collection. Future studies should employ validated multi-item instruments or objective measures (e.g., actigraphy or wearable devices) to enhance reliability and validity. Consequently, causal interpretations should be made with caution, and future studies are encouraged Consequently, causal interpretations should be made with caution, and future studies are encouraged to employ randomized controlled designs to confirm these findings. Furthermore, subsequent research could benefit from larger longitudinal designs and the inclusion of additional objective indicators, such as salivary cortisol levels. It would also be valuable to explore the integration of mindfulness with complementary interventions, including physical activity or cognitive coping strategies. In addition, incorporating repeated physiological assessments throughout the semester may help disentangle intervention effects from the temporal dynamics of academic stress, which appears to be a central moderating factor in this study.

From a practical standpoint, the results suggest that mindfulness interventions may be particularly effective for enhancing emotional aspects such as loneliness and fostering psychological resilience. However, to more effectively target perceived stress and academic performance, these programs may need to be adapted or combined with complementary strategies (McBride and Greeson, 2023; Alvarado-García et al., 2025). Educational institutions could consider incorporating structured mindfulness-based programs into their student wellness services, particularly at the beginning of the semester before stress levels peak. Integrating these interventions with workshops on sleep hygiene, cognitive coping skills, and physical activity may amplify both psychological and psychophysiological benefits and strengthen students’ capacity to navigate academic demands throughout the semester.

Furthermore, implementing mindfulness-informed sessions during orientation and offering follow-up workshops during high-pressure periods (e.g., examination weeks) may help students manage stress more effectively, improve emotional regulation, and support healthier lifestyle behaviors such as improved sleep, increased physical activity, and more adaptive autonomic functioning. Beyond student-focused programming, training faculty and student-support staff in mindfulness principles could contribute to a more supportive, stress-aware academic environment. Such an institutional climate may reinforce the benefits of individual participation in mindfulness programs, promote a greater sense of belonging, and inform research-based policies tailored to improving students’ mental health, social connectedness, and academic success.

## 5 Conclusion

In conclusion, the results of this study indicate that the mindfulness-based intervention produced modest but meaningful effects in certain psychological and physiological domains, while its overall influence on broader biomedical variables and academic performance remained limited. Notably, the intervention was associated with an increase in conscientiousness and a reduction in feelings of loneliness, reflecting improvements in self-regulatory and socio-emotional functioning. In the autonomic domain, the findings revealed discrete and heterogeneous changes, including small reductions in heart rate accompanied by slight decreases in short-term HRV indices (rMSSD and SD2), suggesting a mixed and context-dependent physiological adjustment rather than a clear enhancement of vagal activity. However, the intervention did not produce significant effects on perceived stress, anxiety, or depressive symptoms, nor did it lead to substantial changes in physical activity or sleep patterns. Taken together, these results suggest that while mindfulness training may foster specific psychological benefits and subtle autonomic modulations, its overall impact appears limited in scope and magnitude within a high-stress academic environment. Future studies with larger and more balanced samples, extended follow-up periods, and multimodal interventions combining mindfulness with behavioral or lifestyle strategies are recommended to more fully elucidate the mechanisms and optimize the efficacy of such programs in university contexts.

## Data Availability

The datasets presented in this study can be found in online repositories. The names of the repository/repositories and accession number(s) can be found in the article/supplementary material.
