# Correction: Autonomic and psychophysiological effects of a 13-week mindfulness-based intervention in university students

**DOI:** 10.3389/fpsyg.2026.1805189

**Published:** 2026-02-19

**Authors:** Juan Camilo Benítez-Agudelo, Eduardo Navarro-Jiménez, Vicente Javier Clemente-Suárez

**Affiliations:** 1 Departamento de Ciencias Sociales, Corporacion Universitaria de la Costa, Barranquilla, Colombia; 2 Faculty of Medicine, Health and Sports, Universidad Europea de Madrid, Villaviciosa de Odón, Madrid, Spain; 3 Universidad Simón Bolívar, Facultad de Ciencias de la Salud, Centro de Investigaciones en Ciencias de la Vida, Barranquilla, Colombia

**Keywords:** academic performance, heart rate variability, loneliness, mindfulness, university students

In the published article, author Eduardo Navarro-Jiménez was incorrectly affiliated with ^1^Departamento de Ciencias Sociales, Corporación Universitaria de la Costa, Barranquilla, Colombia. The correct affiliation is 3Universidad Simón Bolívar, Facultad de Ciencias de la Salud, Centro de Investigaciones en Ciencias de la Vida, Barranquilla, Colombia.

In addition, there was an error in affiliation 2. It was originally listed as *Faculty of Sports Sciences, Universidad Europea de Madrid SLU, Madrid, Spain*. The correct affiliation is Faculty of Medicine, Health and Sports, Universidad Europea de Madrid, Villaviciosa de Odón, Madrid, Spain.

The original version of this article has been updated.

